# Primary immunodeficiencies of the B Lymphocyte

**Published:** 2010-02-25

**Authors:** A Moise, FA Nedelcu, MA Toader, SM Sora, A Tica, DE Ferastraoaru, I Constantinescu

**Affiliations:** * Transplant Immunology Department, ‘Carol Davila’ University of Medicine and Farmacy, BucharestRomania; ** Centre for Immunogenetics and Virology, Fundeni Clinical Institute, BucharestRomania; *** ‘Nicolae Malaxa’ Clinical Hospital, BucharestRomania

**Keywords:** primary immune disorders, immunodeficiency, hypogammaglobulinemia, humoral immunity, recurrent infections

## Abstract

The immune response consists of two main components: humoral immunity represented by B lymphocytes and cellular immunity maintained by the T lymphocytes. Immunoglobulins, produced by B– lymphocytes, are the main mediators of humoral immunity, and deficiencies at this level affect the body's response to infection. Plasmocytes produce nine antibody izotypes: immunoglobulins G (IgG 1, lgG2, lgG3, lgG4), immunoglobulins M (IgM), immunoglobulins A (lgA1, lgA2), immunoglobulins D (IGD) and immunoglobulins E (IgE). Primary hypogammaglobulinemias are characterized by the occurrence of recurrent infections and, paradoxically, by the occurrence of autoimmune diseases. Characteristic for these diseases is that symptoms occur at 7–9 months after birth, when transplacental antibody titers transmitted from the mother decrease, and the infant's body is unable to synthesize them to normal levels. Primary hypogammaglobulinemias are transmitted genetically, but mutations at the molecular level are still not fully understood. The most common are: Bruton agammaglobulinemia , transient newborn hypogammaglobulinemia, selective immunoglobulin deficiency and variable  common immunodeficiency. Treatment consists of monthly antibiotics and immunoglobulins, depending on antibody titers (except for IgA deficiency).

## Introduction

Primary hypogammaglobulinemias are characterized by the occurrence of recurrent infections and, paradoxically, by the occurrence of autoimmune diseases. Characteristic for these diseases is that symptoms occur at 7–9 months after birth, when transplacental antibody titers transmitted from the mother decrease, and the infant' body is unable to synthesize them to normal levels. Primary hypogammaglobulinemias are transmitted genetically, but mutations at the molecular level are still not fully understood. The most common are: Bruton agammaglobulinemia, transient newborn hypogammaglobulinemia, selective immunoglobulin deficiency and variable common immunodeficiency.

## Bruton agammaglobulinemia

### Pathophysiology 

Bruton agammaglobulinemia is a primary immunodeficiency caused by the existence of mutations in the gene that encodes Bruton tyrosine kinase (BTK) on chromosome X. Approximately one third of the mutations are at sites CGG, which encodes for arginine [1, 2]. This disorder was first described by Bruton in 1952 and is a defect in maturation of pre–B lymphocytes in mature B lymphocytes. Thus, plasmocytes are absent and reticuloendhotelial tissue and lymphoid organs (tonsils, spleen, Peyer plaques, lymphnodes) are poorly developed. Immunoglobulin titers are more reduced or absent. The disease occurs with a frequency of approximately 1:250.000 males. Females are only carriers and show no clinical symptoms. The disease signs occur when transplacental IgG antibodies, transmitted from the mother, decrease and due to the plasmocytes' absence cannot synthesize other immunoglobulins. 

### Clinical signs 

First symptoms appear at less than 1–year of age, patients presenting recurrent otitis, sinusitis, pneumonia with encapsulated bacteria such as Streptococcus pneumoniae, Haemophillus influenzae, Pseudomonas aeruginosa, Mycoplasma catarrhalis, Neisseria meningitidis, but also with cutaneous symptoms (impetigo, abscesses, furuncles) caused by group A streptococcus and Staphylococcus aureus. Patients with Bruton's disease are predisposed to enteroviral infections, meningitis, bacterial diarrhea (Campylobacter jejuni) and Giardia infections. In adult patients, obstructive and restrictive pulmonary impairment occurs as a complication of recurrent infections. The incidence of autoimmune diseases (thrombocytopenia, neutropenia, hemolytic anemia, rheumatoid arthritis) is, also, increased. 

### Diagnosis 

IgG titers are low and a value below 100 mg/dl is suggestive for X–linked hypogammaglobulinemia. Confirmation is made by flowcytometry which determines B and T lymphocyte levels. Imagistic studies may suggest the presence of chronic sinus and lung infections and quantitative reduction of lymphoid tissue. Since they were discovered, 5 years ago, spirometry tests have been indicated.[3,4] 

### Treatment

There is not a curative treatment. Therapeutic measures consist of intravenous immunoglobulins (400–600 mg/kg monthly in order to maintain the IgG levels at 500–800 mg/dl), specific treatment of bacterial infections with antibiotics and bronchodilators. Nutritional multivitamins supplement is also recommended. 

### Prognosis and complications

The prognosis is well on a long time basis if the patients are diagnosed in due time and an appropriate therapy with i.v. immunoglobulins is applied, before the appearance of recurrent infectious sequelae. It is important that before surgery, patients with X– linked hypogammaglobulinemia should receive intravenous immunoglobulins. Immunization with living virus vaccine is contraindicated. [5, 6]

## Transient newborn hypogammaglobulinemia 

### Pathophysiology 

At birth, the child is endowed with immunoglobulins from the mother. These are IgG, IgM and IgA levels which are very low. Any infection considerably increases the IgM level. Immunoglobulins from the mother are metabolized in about 3–5 months. Normally, up to 6 months of life, the child synthesizes about 33% of IgG levels, 30% of IgA and 70% of IgM. 

IgG production begins only after 2 months of life and IgA and IgM production even later. Therefore, any delay or extent of physiological hypogammaglobulinemia between the 3rd and the 6th month of life, and of recovery period between 18 and 36 months defines transient newborn hypogammaglobulinemia and is self–limited. Actually, this is a delay of normal IgG synthesis, which normally resolves by itself until the 16th and the 30th month of life. Sometimes, an impairment of IgA or IgM levels is observed. Most cases are spontaneously solved before 3 years old.[7] 

### Clinical signs

Symptoms may be absent or sinopulmonary infections may exist, severe infections being rare. 

### Treatment 

Treatment consists of antibiotics and, in severe cases, a substitution therapy with immunoglobulins could be necessary. 

## Disgammaglobulinemia or selective immunoglobulins deficiency. Selective IgA deficiency

There are several immunological diseases associated with immunoglobulin deficiency, and the most common is selective IgA deficiency. IgA was first described by Graber and Williams in 1952, the first case of IgA deficiency being described 10 years later. The incidence of disease is approximately 1:2.000 individuals, and symptoms appear in 1:500–700 affected people. In this disorder, plasmocytes are unable to produce IgA, although B–lymphocytes level is normal. It seems that there is a lack of B lymphocyte response to interleukins–IL–4, IL–6, IL–7 or IL–10. This disorder may be associated with one or more immunoglobulin deficiencies, particularly of some IgG subclasses deficiency, and the lack of response to immunization with pneumococcal vaccine. Subsequently, some patients develop common variable hypogammaglobulinemia.[8, 10] The use of drugs such as D–penicillamine, sulfasalazina, captopril, valproic acid, carbamazepine, ibuprofen, and exposure to certain infections (rubella, cytomegalovirus [CMV], toxoplasmosis) can cause reversible IgA deficiency. Seasonal variations of IgA antibodies was also described, these increasing in winter. 

### Clinical signs

In a major IgA deficiency, the symptomatology is represented by repetitive sinopulmonary infections, otitis, meningitis and pneumonia. Patients may also experience asthma, allergies and gastrointestinal and urinary infections. People with total IgA deficiency develop antibodies against IgA. In case of blood transfusions, these patients can develop anaphylactic shock reactions. In addition, there is a risk of autoimmune diseases such as autoimmune thrombocytopenia, rheumatoid arthritis, and lupus erythematosus. 

### Diagnosis 

Normal titers of IgA are of 100–400 mg/dl and, in a patient with selective IgA immunodeficiency, they get less than 7 mg/dl. IgA serum which is less than 0.05 g/dl in at least 2 determinations, undetectable secretory IgA and exclusion of other primary and secondary immunodeficiencies focus on diagnosis. Imagistic and pulmonary functional tests help to determine various infections and complications. The treatment is the prevention of infections and proper administration of antibiotics.

## Selective IgG deficiency

It can be classified depending on IgG subclasses deficiency: 60–70% is IgG1, 20–30% IgG2, 5–8% IgG3 and 1–3% IgG4. IgG1 and IgG3 titers reach normal values at 5–7 years old, while IgG2 and IgG4 grow slowly and reach these values at the age of 10 years old. IgG1 and IgG3 are antibodies involved in antitoxins protection (diphtheria toxin, tetanus) and in antiviral protection. Instead, antipolysaccharide antibodies are predominantly IgG2.

### Clinical signs

Most frequently, patients develop recurrent otitis, infection that may progress to deafness or total loss of hearing. Pulmonary infection may develop into an obstructive pattern. Most times, IgG deficiency (especially IgG2 and IgG4) is associated with IgA deficiency and these patients do not respond to immunization with polysaccharide vaccine against pneumococcal or H. influenzae. [12, 13] Moreover, the deficiency of these two subclasses of IgG associated with IgE deficiency meet in a disease called ataxia–teleangiectazie. 

### Treatment 

The treatment consists of individualized antibiotics schemes up to well– documented bacteriological diagnosis, and, in some cases, immunoglobulins substitution is required.

## Selective IgM deficiency

It is a very rare disease (less than 300 cases described in literature) and is defined by low levels of IgM – less than 20 mg/dl in children and less than 2 standard deviations relative to the adult corresponding values. Infectious agents in children are represented by Pneumocystis carinii, Giardia, S. aureus, Salmonella sp, N. meningitidis, CMV, Pseudomonas aeroginosa and Moluscum contagiosum. The agents that cause recurrent infections such as dermatitis, diarrhea, meningitis, respiratory infections, sepsis and even death characterize varicella zooster. [14] 

Treatment consists of immunoglobulin administration. 

## Common variable immunodeficiency

Common variable immunodeficiency (CVID) is characterized by a low titer of immunoglobulin, leading to increased susceptibility to infection. In most patients, the diagnosis is established in the 2^nd^ or 3^rd^ decade of life, but recurrent infections are common since childhood. Incidence of this pathology is about 1:10.000–50.000, without predilection for a particular race and both sexes are equally affected.

### Pathophysiology

The primary immunological defect lies in the inability of B–lymphocytes to differentiate into plasma cells, resulting in a low antibody titer. Genetic abnormalities underlying this disease are the shortage of CD 19 (with a role in regulating the B lymphocytes response to antigens) and mutations in certain genes that encode factors involved in the production of different antibody subclasses and in the production of IL–10, IL–2, IL–4, IL–5, IL–13 (with a role in the cooperation between B and T lymphocytes). Studies have demonstrated a low expression of CD40 ligand in the activated CD4 T lymphocytes, resulting in low production of IL–2. Thus, patients with CVID present low levels of IgG and IgA and approximately 50% of patients present low levels of IgM.

### Clinical signs

Most patients present chronic respiratory tract disorders: sinusitis, otitis, laryngitis, pneumonia. Due to the low IgG production, the most commonly pathogens involved are represented by encapsulated bacteria such as Strptococcus pneumoniae and H. influenzae. Moreover, patients may also present infectious diarrhea (Giardia lamblia, Salmonella, Shigella, Campylobacter) or diarrhea resulted from ulcerative colitis or Crohn's disease. Studies show an increased incidence of gastric adenocarcinoma, lymphoma, as well as benign lymphoproliferative disease (splenomegaly, diffuse lymphadenopathy). Approximately 20% of patients with CVID develop autoimmune diseases, such as autoimmune hemolytic anemia, autoimmune thrombocytopenic purpura, arthritis, thyroiditis. [17, 18]

### Diagnosis 

The diagnosis is both clinical and paraclinical, by emphasizing low IgA, IgG, IgM titers in the absence of other known causes of antibody deficiency. Measuring the levels of IL–2, IL–4, IL–5, IL–6, TNF is an alternative way of targeting the diagnosis, in a patient with recurrent infections. The most frequent diagnosis is made by excluding other immune deficiency problems, whose primary genetic causes are well known. 

### Treatment 

Treatment consists of intravenous or subcutaneous immunoglobulin administration, every 2–4 weeks, in order to maintain the normal antibodies titers (400-500 mg/dl). [19, 20] Most patients respond well to therapy with immunoglobulins except for the patients with gastrointestinal events. Corticosteroids or other immunosuppressants are necessary in the cases of patients with gastrointestinal events and those with severe autoimmune phenomena. Antibiotic therapy should be initiated at the first signs of infection, but long–term prophylaxis is contraindicated because of the risk of antibiotic resistance. 

## Clinical conclusions

Hypogammaglobulinemias are relatively rare, often associated with acute or chronic recurrent infections, resulting in a decreased quality of life and a decreased life expectancy. 

It is necessary to consider these diseases in advance whenever a child or adult presents a recurrent infection after proper antibiotics administration. Next, it is important to determine the causes that lead to insufficient production of differentantibodies classes, to develop specific markers for diagnosis and to redefine treatment protocols in order to decrease the risk of complications.
